# Effects of an omega-3 fatty acid-enriched lipid emulsion on eicosanoid synthesis in acute respiratory distress syndrome (ARDS): A prospective, randomized, double-blind, parallel group study

**DOI:** 10.1186/1743-7075-8-22

**Published:** 2011-04-08

**Authors:** Joan Sabater, Joan Ramon Masclans, Judit Sacanell, Pilar Chacon, Pilar Sabin, Mercè Planas

**Affiliations:** 1 Critical Care Department, Vall d'Hebron University Hospital. Vall d'Hebron Research Institute. Passeig Vall d'Hebron 119-129. 08035 Barcelona, Spain; 2 Biochemistry Department, Vall d'Hebron University Hospital. Vall d'Hebron Research Institute. 119-129. 08035 Barcelona, Spain; 3 Pharmacy Department, Vall d'Hebron University Hospital. Vall d'Hebron Research Institute. 119-129. 08035 Barcelona, Spain; 4 Nutritional Support Unit, Vall d'Hebron University Hospital. Vall d'Hebron Research Institute. 119-129. 08035 Barcelona, Spain; 5 Departament de Medicina, Universitat Autonoma de Barcelona, Spain

## Abstract

**Background:**

The use of lipid emulsions has been associated with changes in lung function and gas exchange which may be mediated by biologically active metabolites derived from arachidonic acid. The type and quantity of the lipid emulsions used could modulate this response, which is mediated by the eicosanoids. This study investigates the use of omega-3 fatty acid-enriched lipid emulsions in ARDS patients and their effects on eicosanoid values.

**Methods:**

Prospective, randomized, double-blind, parallel group study carried out at the Intensive Medicine Department of Vall d'Hebron University Hospital (Barcelona-Spain). We studied 16 consecutive patients with ARDS and intolerance to enteral nutrition (14 men; age: 58 ± 13 years; APACHE II score 17.8 ± 2.3; Lung Injury Score: 3.1 ± 0.5; baseline PaO_2_/FiO_2 _ratio: 149 ± 40). Patients were randomized into two groups: Group A (n = 8) received the study emulsion Lipoplus^® ^20%, B. Braun Medical (50% MCT, 40% LCT, 10% fish oil (FO)); Group B (n = 8) received the control emulsion Intralipid^® ^Fresenius Kabi (100% LCT). Lipid emulsions were administered for 12 h at a dose of 0.12 g/kg/h. We measured LTB_4_, TXB_2_, and 6-keto prostaglandin F_1α _values at baseline [immediately before the administration of the lipid emulsions (T-0)], at the end of the administration (T-12) and 24 hours after the beginning of the infusion (T 24) in arterial and mixed venous blood samples.

**Results:**

In group A (FO) LTB_4_, TXB_2_, 6-keto prostaglandin F_1α _levels fell during omega-3 administration (T12). After discontinuation (T24), levels of inflammatory markers (both systemic and pulmonary) behaved erratically. In group B (LCT) all systemic and pulmonary mediators increased during lipid administration and returned to baseline levels after discontinuation, but the differences did not reach statistical significance. There was a clear interaction between the treatment in group A (fish oil) and changes in LTB_4 _over time.

**Conclusions:**

Infusion of lipids enriched with omega-3 fatty acids produces significant short- term changes in eicosanoid values, which may be accompanied by an immunomodulatory effect.

**Trial registration:**

ISRCTN63673813.

## Background

The lipid emulsions administered in parenteral nutrition (PN) of critically ill patients are rich in long chain triglycerides (LCT), particularly linoleic acid (18:2 n-6), so as to ensure optimum energy intake and to prevent essential fatty acid deficiency [1]. However, in addition to their adverse metabolic effects, these fatty acids may alter pulmonary gas exchange due to their potential pro-inflammatory properties, particularly in high risk patients such as those with respiratory failure [2-5].

ARDS is a clinical condition characterized by acute severe respiratory failure, with an estimated incidence of 200,000 cases/year in the US. Despite recent advances in overall support, ARDS still presents high comorbidity and mortality rates of over 40% [6-8].

A large number of factors that might be involved in the genesis of ARDS have been identified, such as several vasoactive substances which promote cell aggregation and modify permeability. Histamine, serotonin, the cytokine system, and some lipid mediators (prostaglandins, leukotrienes and platelet activating factor - PAF) are some of these factors.

Linoleic acid is the precursor of arachidonic acid and therefore indirectly the precursor of the arachidonic acid-derived lipid mediators known as eicosanoids [9]. Some eicosanoids are inflammatory mediators with high biological activity and are associated with a large number of disease processes, both acute (ARDS, acute pancreatitis, etc.), and chronic (asthma, rheumatoid arthritis, Crohn's disease, etc.) [10-13].

Several strategies have been designed to minimize linoleic acid intake and its possible adverse effects, among them the administration of emulsions with mixtures of long-chain and medium-chain fatty acids (LCT-MCT), or of emulsions enriched with olive oil or with n-3 fatty acids (FO). This latter option is potentially the most interesting; although the administration of FO via the enteral route has been widely studied, the parenteral route in patients with ARDS has not been explored in depth [5,14-17].

Whereas linoleic acid synthesizes series-2 and series-4 eicosanoids via the arachidonic pathway, the n-3 fatty acids synthesize the series-3 eicosanoids, which have a much lower biologically active profile than mediators derived from linoleic acid [18].

Many patients with ARDS have digestive intolerance. We believe that ARDS patients who tolerate parenteral nutrition might benefit from a lipid emulsion enriched with FO administered as lipid intake.

The present study explores whether parenteral administration of a lipid emulsion with a lower amount of linoleic acid and enriched with FO fatty acids can modulate the synthesis of biologically active eicosanoids in patients with ARDS.

## Methods

We studied 16 consecutive patients with ARDS in the first 48 hours after admission, enrolled after approval of the clinical trial by the Vall d'Hebron University Hospital Clinical Trials Committee. The inclusion criteria were: sudden onset of bilateral pulmonary infiltrates in the chest x-ray; PaO_2_/FiO_2 _ratio ≤ 200; pulmonary capillary pressure ≤ 18 mm Hg [6] and requirement of PN according to the recommendations of the American Society of Parenteral and Enteral Nutrition [19].

Exclusion criteria were: age < 18 or > 85 years, pregnancy, liver failure, HIV+, leukopenia (<3500 mm^3^), thrombocytopenia (<100000 mm^3^), severe renal failure (creatinine >6 mg/dl) or requirement of renal dialysis, history of organ transplant, multiple transfusions, participation in other clinical studies simultaneously or in the last 60 days, nitric oxide treatment, corticoids (prednisolone dose greater than 2 mg/kg/d or equivalent), multi-organ failure, severe dyslipemia or treatment with propofol.

The patients were sedated with morphine, midazolam and muscle relaxants if required. All patients were mechanically ventilated (7200 series Mallinckrodt Puritan Bennett, Carlsbad, Calif., USA) in control mode. They were monitored with continuous electrocardiography, heart rate, arterial oxygen saturation using pulse oximetry, invasive arterial pressure and a pulmonary artery catheter with continuous cardiac output by thermodilution.

### Determination of eicosanoids

We studied the thromboxane B_2 _(TXB_2_) cyclooxygenase pathway, a stable metabolite of TXA_2_, and 6-keto prostaglandin F_1_(6-keto PGF_1α_) a stable metabolite of prostacyclin. We also studied leukotriene B_4 _(LTB_4_) of the lipoxygenase pathway.

The eicosanoids were analyzed in arterial blood (post-pulmonary), by arterial access (femoral or radial) and in mixed venous blood (pre-pulmonary) by means of a Swan-Ganz catheter, by enzyme immunoassay (EIA); Amersham International (Buckinghamshire, UK).

The reference values measured in the same laboratory in healthy individuals were used as the normal range.

### Study Design

The patients were randomly assigned to different groups within the first 48 hours of ARDS diagnosis and before receiving artificial nutrition. Group A patients (n = 8, emulsion study group) received Lipoplus^® ^20% B. Braun Medical (50% MCT, 40% LCT, 10% FO). Group B (n = 8, control group) received Intralipid^® ^20% Fresenius Kabi (100% LCT). The lipid emulsions were administered for 12 hours at 0.12 g/kg/h.

We measured concentrations of eicosanoids (TXB_2_, 6-ketoPGF_1α _and LTB_4_) in the arterial and mixed venous samples at baseline [immediately before the administration of the lipid emulsions (T-0)], at the end of the administration (T-12), and 24 hours from the start of the infusion (T-24).

### Statistical analysis

The data were imported from Access databases to SPSS. Means and standard deviations were calculated for numerical variables. Differences in the means were analyzed by the T-test. Differences in time of the parameters studied, depending on the type of emulsion, were assessed by the Wilcoxon test. We also studied the interaction between treatment and all the eicosanoids using ANOVA. The level of statistical significance was defined as p < 0.05.

## Results

### Characteristics of the studied population

The baseline characteristics are shown in Table 1. No statistically significant differences were seen in the baseline values.

**Table 1:** Clinical characteristics of patients

Diagnosis	Sex	Age	PaO_2_/FiO_2_	LIS	APACHEII	PEEP	Outcome
**Group A: FO**							

1. Pneumonia	M	74	98	4	18	7	D

2. Pneumonia	M	48	166	2.3	17	8	D

3. Pneumonia	M	64	133	3.6	18	12	D

4. Sepsis	M	50	189	3	15	10	L

5. Smoke aspiration	M	40	132	3	16	11	L

6. Bronchoaspiration	M	72	84	2.6	21	8	D

7. Mediastinitis	M	70	147	3.3	19	10	L

8. Bronchoaspiration	M	36	183	2.5	15	7	L

**Total Group A:**	**8 M**	**56 ± 15**	**141 ± 37**	**3 ± 0.5**	**17.4 ± 2.1**	**9.1 ± 1.9**	**4D/4L**

**Group B: LCT**							

1. Pancreatitis	F	52	152	3	16	11	L

2. Bronchoaspiration	M	57	198	4	21	15	L

3. Bronchoaspiration	M	56	177	2.6	17	9	D

4. Status postpneumectomy	M	63	60	4	19	10	L

5. Pneumonia	M	71	181	3	18	10	L

6. Pneumonia	M	43	186	3.3	14	8	L

7. Pneumonia	M	53	153	3	19	10	D

8. Pneumonia	F	81	157	3	22	9	L

**Total Group B**	**6M/2F**	**59 ± 12**	**158 ± 43**	**3.2 ± 0.5**	**18.2 ± 2.6**	**10.3 ± 2.1**	**2D/6L**

**Total n:16**	**14M/2F**	**58 ± 13**	**149 ± 40**	**3.1 ± 0.5**	**17.8 ± 2.3**	**9.7 ± 2**	**6D/10L**

LIS: Lung Injury Score. APACHEII: Acute Physiology and Chronic Health Evaluation. PEEP: Positive End Expiratory Pressure. M: male. F: female. D: dead. L: alive.

### Systemic eicosanoid values

Eicosanoid arterial blood values are shown in Table 2. All the values measured in patients with ARDS during the study, both in the arterial and mixed venous samples, were higher than the reference values of the healthy population. We observed significant differences between the three eicosanoid baseline levels, which were higher in Group A (FO) than in Group B.

**Table 2:** (Systemic arterial eicosanoid levels according to the emulsion type administered (values in pg/ml)):

Emulsion A (FO)					Emulsion B (LCT)		
	**Normal Value**	**T(0):Pre infusion** **(Basal)**	**T(12):Post infusion**	**T(24): 24 h from start of infusion**	**T(0):Pre infusion** **(Basal)**	**T(12):Post infusion**	**T(24): 24 h from start of infusion**

**TXB_2_**	<150	362.3 ± 128.9^(**)^	278.5 ± 46.2	291.5 ± 120.6	206.4 ± 66.7^(**)^	470.6 ± 243.2	262.7 ± 97.9

**LTB_4 _**	**<75**	569.3 ± 312.2^(*)(**)^	283.3 ± 145.3^(*)^	210.9 ± 114.6	233.4 ± 147.9^(**)^	365.1 ± 206.2	550 ± 488.2

**6-keto-PGF_1α_**	<50	633.4 ± 218^(**)^	598.1 ± 240	526.9 ± 209.3	412.8 ± 132.8^(**)^	708 ± 239.2	493.4 ± 156

(*) Intragroup significant differences.

(**) Basal values significant differences between both groups.

There were clear differences in the two populations with regard to the behaviour of the eicosanoids. In group A, treated with the FO-enriched emulsion (group A), all eicosanoid values tended to decrease after the administration T(12). Twelve hours after ending perfusion T(24), TXB_2 _levels increased compared with T(12) but were lower than baseline levels T(O), LTB_4 _levels decreased persistently, recording lower levels than T(12), as did 6-keto-PGF_1α_levels, although only LTB_4 _levels presented statistically significant differences.

The opposite trend was seen in the group treated with LCT, in which all the eicosanoid values rose after the administration of the emulsion. Twelve hours after ending perfusion T(24) TXB_2 _decreased compared with T(12) but its levels remained higher than baseline T(0); LTB_4 _levels continued to increase, being higher than at T(12): 6-keto-PGF_1α _decreased with respect to T(12) but remained higher than at baseline T(0).

### Eicosanoid values in mixed venous blood

Eicosanoid values in mixed venous blood were also higher than the reference values in the healthy population. In group A, baseline LTB_4 _levels were significantly higher.

The eicosanoid values in mixed venous blood are shown in Table 3. We found changes parallel to those found at peripheral arterial level at the end of lipid emulsion administration T(12). Twelve hours after the end of lipid emulsion administration T(24) in the FO group, TXB_2 _continued to decrease with respect to T(12), as did LTB_4_, 6-keto-PGF_1α _levels increased with regard to T(12) but remained below baseline T(0) levels. In the LCT group at T(24), TXB_2 _decreased with respect to T(12) and fell below baseline levels, LTB_4 _increased with regard to T(12), 6-keto-PGF_1α _decreased with respect to T(12) but remained above baseline; however the differences were small and did not reach statistical significance.

**Table 3:** (Eicosanoid levels in mixed venous blood according to the emulsion type administered (values in pg/ml)):

Emulsion A (FO)				Emulsion B (LCT)		
	**T(0):Pre infusion** **(Basal)**	**T(12):Post infusion**	**T(24): 24 h from start of infusion**	**T(0):Pre infusion** **(Basal)**	**T(12):Post infusion**	**T(24): 24 h from start of infusion**

**TXB_2_**	375.4 ± 146.3	305.4 ± 70.3	279.6 ± 127.1	283.3 ± 123.4	326.1 ± 132.4	231 ± 88.9

**LTB_4_**	433 ± 186.2^(**)^	239.6 ± 130.8	234.4 ± 111.8	200.3 ± 160.7^(**)^	260.6 ± 150.5	342.6 ± 230.4

**6-keto-PGF_1α_**	660.1 ± 298.9	529.7 ± 178.3	566.4 ± 224.8	509.5 ± 134.6	746.4 ± 532.4	525.5 ± 298.3

(**) Basal values significant differences between both groups.

Applying the ANOVA test, we observed a clear interaction between the treatment in group A (fish oil) and changes in LTB_4 _over time with p < 0.001 (systemic LTB_4_) and p < 0.01 (mixed venous blood).

## Discussion

All the ARDS patients in our series presented higher baseline values of eicosanoids, in both systemic arterial and mixed venous blood, than the healthy population. On administering the lipid emulsions at 0.12 g/kg/h for 12 hours, patients who received the LCT-rich emulsion presented increased values in all eicosanoids at the end of the infusion compared with baseline. Twelve hours after the end of the emulsion, TXB_2 _and 6-keto-PGF_1α _levels decreased and LTB_4 _levels, both systemic and pulmonary, continued to increase. As for the patients who received FO enriched lipid emulsion, all eicosanoid values tended to decrease at the end of administration, at both systemic and pulmonary level. Twelve hours after the end of the administration the behaviour of the mediators was more erratic. The only statistically significant difference was found in the LTB_4 _values at systemic level.

We observed significant differences in baseline eicosanoid levels, which were higher in the FO group, especially in the arterial blood sample. It is difficult to evaluate these baseline differences in a population which was otherwise totally homogeneous, but we think that they may be due to the major interindividual eicosanoid variability observed in critically ill patients. This makes it even more important to use a point by point calculation instead of ANOVA, because the further away from time 0, the more the circumstances may modify the result and invalidate the conclusions.

The erratic behaviour of the lipid mediator 12 hours after the end of the lípid emulsions may be due to the loss of beneficial effect of FO and the increase in the detrimental effect of LCT over time.

In this trial we focused on the effect of lipid emulsion on eicosanoid synthesis. Hemodynamic and gas exchange variations were discussed in a previous study in which we did not observe any significant variation in gas exchange, pulmonary mechanical parameters, or in systemic and arterial hemodynamics [20], in contrast to another recent report with a similar lipid emulsion [21].

Besides its gas exchange function, the lung is involved in both the synthesis and catabolism of several inflammatory mediators [22]. We know that it is mainly responsible for eicosanoid synthesis of PGI_2 _and TXA_2_, which are considered the most important derivatives of arachidonic acid in the lung. TXA_2 _may increase lung vascular resistance, whilst prostacyclin dilates the vascular bed. The LTB_4 _released by the alveolar macrophages and neutrophils is heavily involved in the inflammatory response and facilitates capillary extravasation [23]. Several authors suggest that leukotrienes may play a crucial role in the development of ARDS after the initial cell injury [24]. Furthermore, the leukotrienes, and particularly LTB_4_, play an important role in pulmonary leukocyte chemotaxis which, in turn, could trigger the rest of the inflammatory cascade [25,26]. In fact, increased concentrations of leukotrienes have been found in the bronchoalveolar lavage and plasma in patients with lung injury, as well in those at risk of developing one [27,28].

Several studies have associated systemic levels of LTB_4 _with the severity of the ARDS and the degree of hypoxemia. It has also been reported that the higher the plasma levels of LTB_4_, the higher the degree of hypoxemia. Increased mortality has been observed in patients with higher baseline levels of plasma LTB_4 _[26,29]. All these data confirm that LTB_4 _is a proinflammatory molecule with a fundamental role in the cascade that leads to the genesis and evolution of respiratory distress, as well as being an excellent prognostic marker [25,27].

Attempts have been made to modulate the inflammatory response in ARDS using various strategies (corticoids, indomethacin, etc..) without much success in humans [30,31]. One enormously attractive approach would be to modulate the release of final eicosanoid mediators by modifying the intake of their potential initial precursors. PN rich in LCT, and in particular in linoleic acid, is known for its potential effects on the synthesis of proinflammatory mediators and secondarily, a potential deterioration in lung function [32,33] - as we saw in our study in our LCT patients, who presented significant increases in all eicosanoid values. Several strategies have been used to minimize the intake of linoleic acid and to avoid its negative effects. The use of emulsions with LCT/MCT would lead to the synthesis of lower amounts of eicosanoid, since the rapidly oxidized MCT would not act as eicosanoid precursors. Many studies in the literature report clinically divergent results [14,17,34-36]. Of course, MCT intake may induce ketosis, with the limitations that this presents in patients with diabetes mellitus or in similar situations [37]. Comparing patients with ARDS treated with LCT vs. LCT/MCT emulsions, our group reported neutral results in eicosanoid synthesis [38]. A lower amount of PUFA n-6 is provided in LCT/MCT emulsions, but the n-6/n-3 ratio is not changed, with the result that non-recommendable values may theoretically persist. Enriching lipid emulsions with FO, which could "modulate" the response of the body to aggression, has been studied as a treatment strategy in patients with ARDS receiving enteral nutrition [16], but there is little information available on patients with intolerance of the digestive route and who require PN.

The use of OF-enriched PN has been shown to be safe and well tolerated in critical patients [20,39]. There are also many examples of a favourable profile in eicosanoid synthesis, particularly of LTB_4_, in many chronic diseases such as psoriasis [40]. But where it has been studied most is in the surgical patient. In one series of surgical patients the use of an emulsion consisting of LCT-MCT-FO and olive oil minimized the production of LTB_4 _and increased LTB_5_, and reduced hospital stay [41]. An immunomodulatory effect on the generation of lipid mediators has also been found after administering an FO-enriched emulsion [42]. This same favourable immunomodulatory effect could be observed in septic patients [43].

Focusing on the effect of FO-enriched lipid emulsions on acute lung injury (ALI) and ARDS, we found many studies of animal models but very few of humans. A protective effect of n-3 has been reported in ALI animal models; the administration of arachidonic acid (AA) or eicosapentanoic acid (EPA) aggravates or reduces, respectively, the edema in animal models (rabbits) with respiratory failure in sepsis [44]. Administration of AA is accompanied by a higher release of leukotrienes and TXA_2_, whereas with the administration of EPA LTB_4 _formation is blocked and there are increases in n-5 series and TXA_3 _[43-48]. The FO-rich lipid emulsions probably reduce the adverse effects of the synthesis of mediators by the AA pathway, such as pulmonary hypertension, pulmonary edema and the accumulation of pulmonary neutrophils [43]. However, little information is available on the possible modulation with FO-enriched lipid emulsions in patients with ARDS. In a recent trial with similar diet in a septic population, an increase in EPA levels was demonstrated with a decrease in cytokines and an improvement in gas exchange [21].

One of the limitations of our trial is the small population size due to our decision to include ARDS patients with low levels of multiorgan dysfunction. Because of our trial design, with short periods of lipid emulsion administration, we cannot draw conclusions regarding either survival or quality of life. The baseline eicosanoid differences were larger in arterial blood samples.

## Conclusions

In our series of patients with ARDS, we found a favourable lipid mediator synthesis profile in patients treated with an FO-enriched lipid emulsion, but not in patients treated with LCT. This potentially significant finding should be confirmed in clinical studies of patients receiving parenteral nutrition with a lipid emulsion similar to the one used here. It is well known that these mediators play a crucial role in this highly "inflammatory" and severe process in patients with ARDS.

## List of Abbreviations

ARDS: Acute respiratory distress syndrome; APACHEII: Acute Physiology and Chronic Health Evaluation; MCT: Medium-chain triglycerides; LCT: Long-chain triglycerides; FO: fish oil; LTB_4_: Leukotriene B_4_; TXB_2_: thromboxane B_2_; 6-keto PGF_1α_: 6-keto prostaglandin F_1_; PN: Parenteral nutrition; n-6: Fatty acids of the n-6 series; n-3: Fatty acids of the n-3 series; TXA_2_: thromboxane A_2_; SPSS: Statistical Package for the Social Sciences; PG I_2_: Prostaglandin I_2_; PUFA n-6: Polyunsaturated series 6 fatty acid; LTB_5_: Leukotriene B_5_; ALI: acute lung injury; AA: arachidonic acid; EPA: eicosapentanoic acid; n-5: Fatty acids of the n-5 series.

## Competing interests

The authors declare that this study has been financed by B. Braun Medical.

## Authors' contributions

JS participated in both the study design and coordination, and drafted the manuscript. JM participated in both the study design and coordination, and performed the statistical analysis. JS participated in both the study design and coordination. PC participated in the study design. PS participated in the study design. MP designed and coordinated the study, and reviewed the final manuscript. All authors read and approved the final manuscript.
